# Examining sleep deficiency and disturbance and their risk for incident dementia and all-cause mortality in older adults across 5 years in the United States

**DOI:** 10.18632/aging.202591

**Published:** 2021-02-11

**Authors:** Rebecca Robbins, Stuart F. Quan, Matthew D. Weaver, Gregory Bormes, Laura K. Barger, Charles A. Czeisler

**Affiliations:** 1 Division of Sleep and Circadian Disorders, Departments of Medicine and Neurology, Brigham and Women’s Hospital, Boston, MA 02115, USA; 2 Division of Sleep Medicine, Harvard Medical School, Boston, MA 02115, USA; 3 Department of Mathematics, Boston College, Boston, MA 02467, USA

**Keywords:** Alzheimer’s disease, preventative health care, sleep, longevity

## Abstract

Background: Sleep disturbance and deficiency are common among older adults and have been linked with dementia and all-cause mortality. Using nationally representative data, we examine the relationship between sleep disturbance and deficiency and their risk for incident dementia and all-cause mortality among older adults.

Methods: The National Health and Aging Trends Study (NHATS) is a nationally-representative longitudinal study of Medicare beneficiaries in the US age 65 and older. Surveys that assessed sleep disturbance and duration were administered at baseline. We examined the relationship between sleep disturbance and deficiency and incident dementia and all-cause mortality over the following 5 years using Cox proportional hazards modeling, controlling for confounders.

Results: Among the sample (n = 2,812), very short sleep duration (≤5 hours: HR = 2.04, 95% CI: 1.26 - 3.33) and sleep latency (>30 minutes: HR = 1.45, 95% CI: 1.03 - 2.03) were associated with incident dementia in adjusted Cox models. Difficulty maintaining alertness (“Some Days”: HR = 1.49, 95% CI: 1.13 - 1.94 and “Most/Every Day”: HR = 1.65, 95% CI: 1.17 - 2.32), napping (“Some days”: HR = 1.38, 95% CI: 1.03 - 1.85; “Most/Every Day”: HR = 1.73, 95% CI: 1.29 - 2.32), sleep quality (“Poor/Very Poor”: HR = 1.75, 95% CI: 1.17 - 2.61), and very short sleep duration (≤5 hours: HR = 2.38, 95% CI: 1.44 - 3.92) were associated with all-cause mortality in adjusted Cox models.

Conclusions: Addressing sleep disturbance and deficiency may have a positive impact on risk for incident dementia and all-cause mortality among older adults.

## INTRODUCTION

Approximately 5.8 million adults in the U.S. are living with Alzheimer’s disease or related dementia, and 16 million are expected to be living with Alzheimer’s disease by 2050 [1]. Both sleep disturbances and abnormal sleep duration, although both often modifiable, are associated with the development and progression of Alzheimer’s disease [2]. Furthermore, recent work suggests that older adults (above age 65) who report signs of good sleep health (i.e., reports of waking and feeling refreshed) demonstrate better cognitive function [3], which may buffer against the development of Alzheimer’s disease and dementia. According to the National Sleep Foundation, there is more sleep disturbance reported among older adults than any other age group [4]. Data collected as part of a prospective observational study among older adults found over 50% of the participants reported at least one sleep difficulty “most of the time,” [5] whereas these complaints were reported by only 20% of younger adults (below age 65) [6].

Sleep disturbance and insufficiency have been shown to be associated with both the development and progression of Alzheimer’s disease and with all-cause mortality [7, 8]. Among a cohort of 737 older adults without dementia, adults with high sleep fragmentation had a 1.5-fold risk of developing Alzheimer’s Disease compared to those with low sleep fragmentation [9]. Similarly, in a prospective analysis, sleep disturbance was linked with incident cognitive impairment [10], while another prospective analysis found sleep disturbance was linked with both incident dementia as well as mortality [11]. In related studies, prospective analyses of 1,951 older adults found that difficulty maintaining alertness was associated with increased risk for dementia [12]; in a cohort of 1,245 older women, longer sleep latency was associated with higher risk of cognitive impairment over an average follow-up period of 4.9 years [13]. Furthermore, compared to cognitively normal individuals, those with either with self-reported obstructive sleep apnea (OSA) diagnosis [14] or physician-diagnosed OSA [15] developed more Alzheimer’s disease biomarkers, such as amyloid-beta plaques or tau proteins, over time compared to those without OSA. In addition, previous research has found associations between both long (>9 hours) and short (<7 hours) sleep duration and Alzheimer’s disease and dementia. Specifically, in research with data from the Framingham Heart Study, self-reports of longer sleep (>9 hours) were associated with all-cause dementia and clinical Alzheimer disease, but self-reports of short sleep (<6 hours) were not [16]. However, according to a meta-analysis of 27 studies, both short (< 7 hours) and long sleep duration (> 8 hours) were both associated with approximately 86% greater risk for Alzheimer’s disease and dementia [7].

Prior research has also examined the association among sleep characteristics, sleep deficiency, alertness and all-cause mortality [17–19]. In a prospective analysis, researchers found that older short (<6 hours) habitual sleepers were at approximately 50% greater risk for all-cause mortality after adjusting for confounders [18]. In research conducted by Chen and colleagues using the Pittsburgh Sleep Quality Index (PSQI), researchers found an association between high PSQI scores (indicative of poor sleep) and all-cause mortality, but this relationship disappeared after controlling for confounders such as depression [19]. In the same study, but with the sub-components of the PSQI (i.e., sleep medication use, sleep quality, and sleep duration), researchers found that those reporting long sleep (>9 hours) were at greater risk for all-cause mortality, but short sleepers were not [19]. Another prospective cohort study found an opposite result. Specifically, Bertisch and colleagues found an association between objectively measured short sleep duration and all-cause mortality, but not long sleep [17]. The same study examined the relationship between those reporting either short or long sleep duration and insomnia in relation to all-cause mortality, but did not find any significant associations [17]. According to a meta-analysis of prospective studies, both short and long sleep were associated with greater risk of all-cause mortality in adults [20, 21]. Another meta-analysis of 24-hour sleep duration found only long sleep (>9 hours) was associated with greater risk of mortality.

Research on sleep disturbance and deficiency and all-cause mortality therefore has shown conflicting results. Further, few studies have included a comprehensive set of sleep characteristics in a single examination of incident dementia and all-cause mortality. We address these gaps in the literature and examine the relationship between sleep disturbance, sleep duration, alertness and incident dementia and all-cause mortality across a five-year time interval using nationally representative data collected among older adults in the U.S.

## RESULTS

Table 1 displays demographic characteristics of the sample at baseline (Year 2013: n = 1,575; Year 2014: 1,237). The average age was 76.9 (s.d. = 7.5 years). The sample was comprised of 60% female respondents (both years) and 72% white respondents (both years), followed by approximately 20% black (2013: 20%; 2014: 19%), 3% Hispanic/Latino (both years), and 6% Asian (both years) respondents. Among respondents, 48% and 44% reported being married in 2013 and 2014, respectively. Nearly 44% and 45% demonstrated clinical depression in 2013 and 2014, respectively. The most common comorbid condition among the sample was cancer (2013: 5%; 2014: 7%), followed by hypertension (2013: 3%; 2014: 2%).

**Table 1:** Demographic characteristics of the study sample who received the sleep questions in years 2013 and 2014 (N = 2,812).

**Variables**	**2013** **(n = 1,575)**		**2014** **(n = 1,237)**	
	**%**	**N**	**N**	**%**
Gender				
Male	613	40.4	496	39.8
Female	904	59.6	749	60.2
Race				
White	1,091	71.9	895	71.9
Black	295	19.5	240	19.3
Hispanic/Latino	45	3.0	31	2.5
Asian	86	5.7	79	6.4
Marital Status				
Married	727	47.9	544	43.7
Living with partner	25	1.7	21	1.7
Separated	19	1.3	26	2.1
Divorced	165	10.9	138	11.1
Widowed	520	34.3	476	38.2
Never married	61	4.0	40	3.2
Chronic Conditions			
Depressed	665	43.8	556	44.7
Heart Attack	41	2.7	37	3.0
Heart Disease	29	1.9	28	2.3
High Blood Pressure	39	2.6	27	2.2
Arthritis	58	3.8	38	3.1
Diabetes	17	1.1	8	0.6
Stroke	40	2.6	37	3.0
Cancer	71	4.7	85	6.8

Table 2 displays descriptive statistics summarizing sleep variables. Approximately 60% of participants reported experiencing difficulty with alertness “never” or “rarely” (2013: 59.63%, 2014: 61.35); nearly one half of participants reported “never” or “rarely” taking naps (2013: 44.09%, 2014: 44.03%). More than half of participants reported taking fewer than 15 minutes to fall asleep (2013: 52.93%, 2014: 52.22%), and more than half reported sleeping 7-8 hours per night (2013: 55.05%, 2014: 54.74%). Nearly 70% of participants reported a sleep quality of “good” or “very good” (2013: 68.74%, 2014: 69.62%), and over 90% of participants reported snoring “never” or “rarely” (2013: 91.87%, 2014: 92.84%).

**Table 2:** Descriptive statistics summarizing 2013 and 2014 demographic variables (N = 2,812).

**Variables**	**2013** **(n = 1,575)**		**2014** **(n = 1,237)**	
	**N**	**%**	**N**	**%**
Difficulty with Alertness				
Never, rarely	901	59.63%	1,722	61.35%
Some days	424	28.06%	735	26.18%
Most, every day	186	12.31%	350	12.47%
Nap Frequency				
Never, rarely	668	44.09%	1,240	44.03%
Some days	429	28.32%	775	27.52%
Most, every day	418	27.59%	801	28.44%
Sleep Latency				
<15 minutes	695	52.93%	1,269	52.22%
15-30 minutes	385	29.32%	720	29.63%
>30 minutes	233	17.75%	441	18.15%
Sleep Quality				
Good, Very Good	1,040	68.74%	1,957	69.62%
Fair	349	23.07%	630	22.41%
Very poor, poor	124	8.20%	224	7.97%
Sleep Duration				
7-8 hours	818	55.05%	1,508	54.74%
≤5 hours	60	4.04%	119	4.32%
6-7 hours	419	28.20%	769	27.91%
≥ 9 hours	189	12.72%	359	13.03%
Snoring				
Never, rarely	1,390	91.87%	2,605	92.84%
Some nights	87	5.75%	143	5.10%
Most, every night	36	2.38%	58	2.07%

### Examining the relationship between each sleep characteristic and incident dementia

Table 3 summarizes results of Cox proportional hazard models examining each sleep characteristic and incident dementia.

**Table 3:** Cox models examining each sleep disturbance characteristic and incident dementia (N = 2,812).

	**Incident dementia**				**Incident dementia**			
	***Unadjusted models***				***Fully adjusted models*^a^**			
	**HR**	***p*-value**	**Lower**	**Upper**	**HR**	***p*-value**	**Lower**	**Upper**
Difficulty with Alertness							
Never/Rarely	Reference							
Some Days	**1.32**	**0.034**	**1.02**	**1.71**	1.13	0.392	0.83	1.43
Most Days/Every Day	1.20	0.286	0.85	1.71	1.01	0.970	0.69	1.46
Nap Frequency								
Never/Rarely	Reference							
Some Days	1.22	0.160	0.92	1.61	1.08	0.631	0.76	1.45
Most Days/Every Day	1.21	0.185	0.94	1.58	0.96	0.786	0.71	1.29
Sleep Latency								
<15 minutes	Reference							
15-30 minutes	**1.41**	**0.017**	**1.06**	**1.89**	1.22	0.192	0.92	1.67
>30 minutes	**1.65**	**0.002**	**1.20**	**2.27**	**1.45**	**0.032**	**1.03**	**2.03**
Sleep Quality								
Good/Very Good	Reference							
Fair	1.08	0.574	0.82	1.43	1.03	0.828	0.73	1.31
Poor/Very Poor	1.01	0.954	0.65	1.58	1.11	0.644	0.65	1.59
Sleep Duration								
7-8 hours	Reference							
≤5 hours	**1.81**	**0.011**	**1.14**	**2.86**	**2.04**	**0.004**	**1.26**	**3.33**
6-7 hours	0.88	0.250	0.62	1.12	0.86	0.330	0.63	1.17
≥ 9 hours	**1.43**	**0.030**	**1.04**	**1.97**	0.97	0.857	0.66	1.39
Snoring								
Never/Rarely	Reference							
Some Nights	**1.62**	**0.024**	**1.06**	**2.54**	1.52	0.079	0.95	2.44
Most Nights/Every Night	0.70	0.479	0.26	1.88	0.39	0.184	0.09	1.56

Note: bold indicates significance at the p<0.05 level.

^a^Adjusted models control for demographic characteristics, including age, marital status, race, education, health conditions, and body weight.

In the unadjusted models, participants who slept both fewer than 5 hours (HR = 1.81, 95% CI: 1.14 - 2.86, p<0.05). And more than 9 hours per night (HR = 1.43, 95% CI: 1.04 - 1.97, p<0.05) demonstrated significantly higher risk for incident dementia. Participants who reported taking between 15 and 30 minutes to fall asleep showed a higher risk for incident dementia (HR = 1.41, 95% CI: 1.06 - 1.89, p<0.05), as did those who took longer than thirty minutes to fall asleep (HR = 1.65, 95% CI: 1.20 - 2.27, p<0.01).

**Figure 1:**
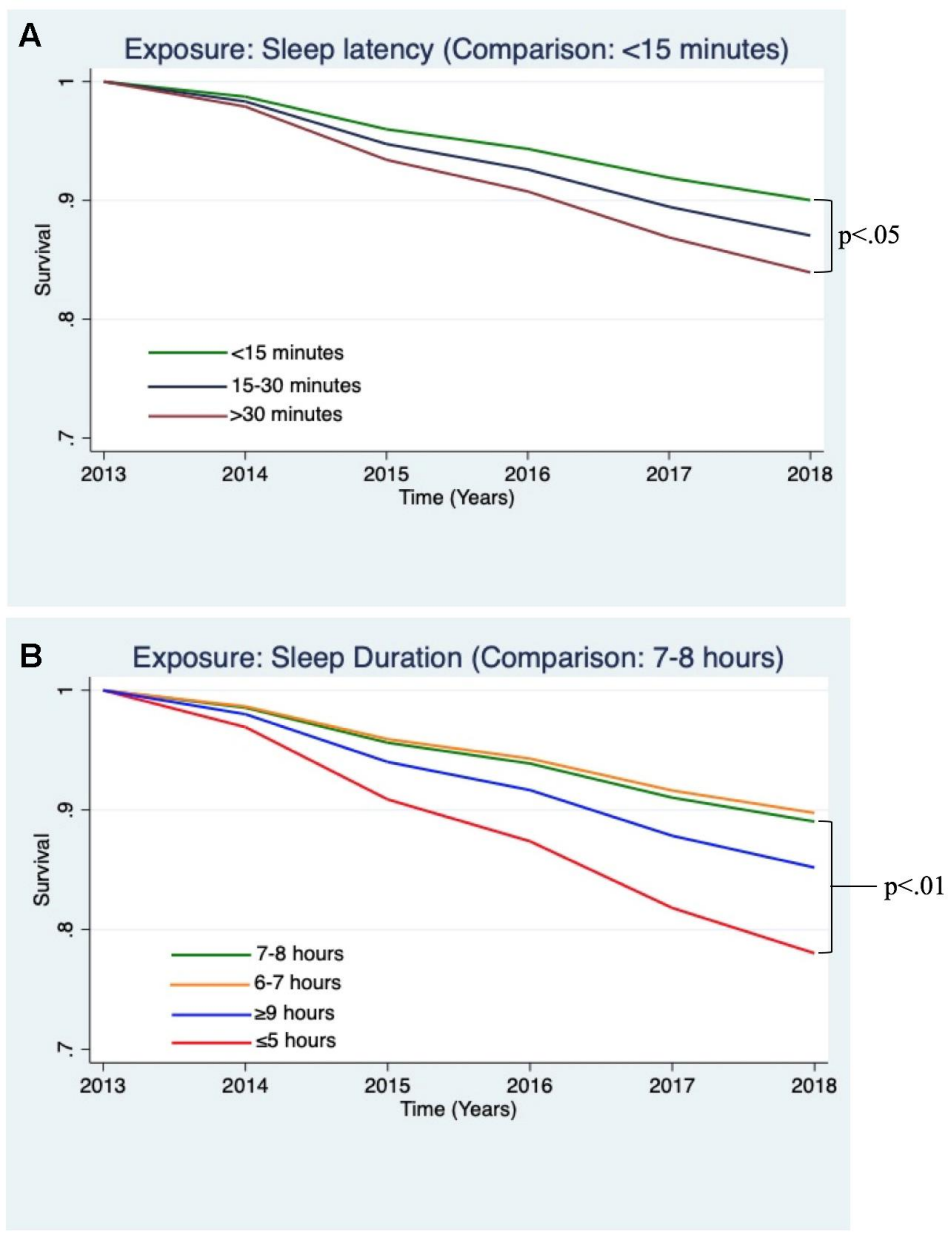
**Estimated survival curves displaying the relationships between sleep variables and incident dementia, adjusting for covariates, which were found to be significant in the Cox hazard proportional models.** (**A**) Survival curve from the Cox model examining incident dementia and sleep latency, adjusting for covariates. Sleep latency >30 minutes, as compared to <15 minutes, was associated a greater risk of incident dementia (p<0.05). (**B**) Survival curve from Cox model examining incident dementia and sleep duration, adjusting for covariates. Sleep duration ≤5 hours, compared to 7-8 hours, was associated a greater risk of incident dementia (p<0.01).

In fully adjusted models, participants who reported taking 30 minutes or longer to fall asleep demonstrated higher risk for incident dementia (HR = 1.45, 95% CI: 1.03 - 2.03, p<0.05, see Figure 1A). Participants who reported sleeping 5 hours or fewer per night demonstrated significantly higher risk for incident dementia (HR = 2.04, 95% CI: 1.26 - 3.33, p<0.01, see Figure 1B).

### Examining the relationship between each sleep variable and all-cause mortality

Table 4 summarizes results of the Cox proportional hazard models examining each sleep characteristic and all-cause mortality. In the unadjusted models, a greater risk for all-cause mortality was associated with self-reported difficulty with alertness both “most/every day” (HR = 2.23, 95% CI: 1.79 - 2.75, p<0.001) and “some days” (HR = 1.50, 95% CI: 1.25 - 1.82, p<0.001). Risk for all-cause mortality was increased for participants who slept for longer than eight hours per night (HR = 2.14, 95% CI: 1.73 - 2.65, p<0.001) and for participants who reported napping “some days” (HR = 1.32, 95% CI: 1.06 - 1.64, p<0.05) or “most/every day” (HR = 2.23, 95% CI: 1.84 - 2.79, p<0.001). Additionally, increased risk of all-cause mortality was associated with self-reported sleep quality of “poor” or “very poor” (HR = 1.33, 95% CI: 1.00 - 1.77, p<0.05).

**Table 4:** Cox models examining each sleep disturbance characteristic and all-cause mortality (N = 2,812).

	**All-cause mortality**				**All-cause mortality**			
	***Unadjusted models***				***Fully adjusted models*^a^**			
	**HR**	***p*-value**	**Lower**	**Upper**	**HR**	***p*-value**	**Lower**	**Upper**
Difficulty with Alertness								
Never/Rarely	Reference							
Some Days	**1.50**	**0.000**	**1.25**	**1.82**	**1.49**	**0.004**	**1.13**	**1.94**
Most Days/Every Day	**2.23**	**0.000**	**1.79**	**2.75**	**1.65**	**0.004**	**1.17**	**2.32**
Nap Frequency								
Never/Rarely	Reference							
Some Days	**1.32**	**0.012**	**1.06**	**1.64**	**1.38**	**0.032**	**1.03**	**1.85**
Most Days/Every Day	**2.23**	**0.000**	**1.84**	**2.79**	**1.73**	**0.000**	**1.29**	**2.32**
Sleep Latency								
<15 minutes	Reference							
15-30 minutes	**1.37**	**0.012**	**1.06**	**1.64**	1.32	0.053	0.99	1.75
>30 minutes	1.20	0.140	0.94	1.54	1.14	0.444	0.81	1.63
Sleep Quality								
Good/Very Good	Reference							
Fair	1.13	0.225	0.93	1.37	1.24	0.119	0.94	1.63
Poor/Very Poor	**1.33**	**0.043**	**1.00**	**1.77**	**1.75**	**0.006**	**1.17**	**2.61**
Sleep Duration								
7-8hours	Reference							
≤5 hours	1.40	0.087	0.95	2.07	**2.38**	**0.001**	**1.44**	**3.92**
6-7 hours	1.05	0.623	0.86	1.29	1.22	0.160	0.92	1.61
≥9 hours	**2.14**	**0.000**	**1.73**	**2.65**	1.31	0.171	0.88	1.94
Snoring								
Never/Rarely	Reference							
Some Nights	**1.41**	**0.037**	**1.02**	**1.96**	1.40	0.181	0.85	2.29
Most Nights/Every Night	0.98	0.959	0.56	1.75	0.71	0.506	0.26	1.92

Note: bold indicates significance at the p<0.05 level.

^a^Adjusted models control for demographic characteristics, including age, marital status, race, education, health conditions, body weight, and incident dementia.

**Figure 2:**
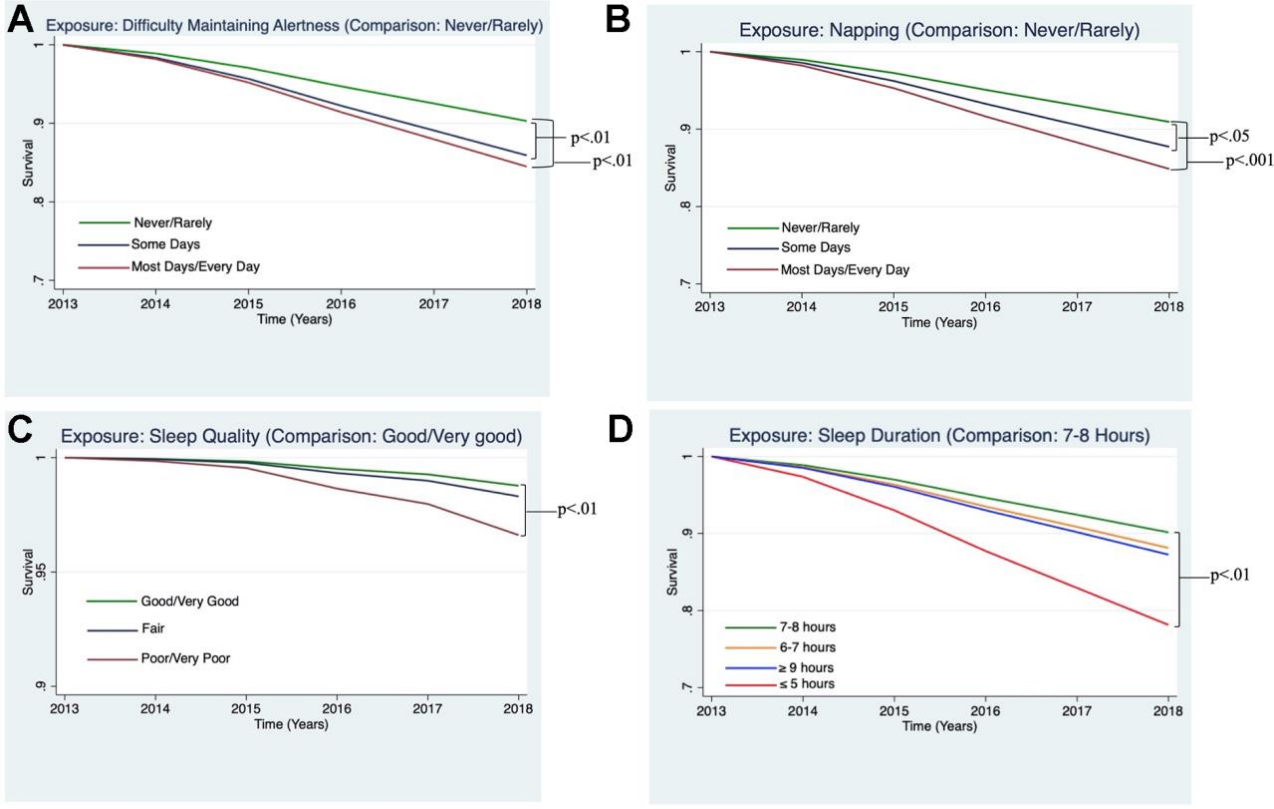
**Estimated survival curves displaying the relationships between sleep variables and all-cause mortality, adjusting for covariates, which were found to be significant in the Cox hazard proportional models.** (**A**) Survival curve from Cox model examining all-cause mortality and difficulty maintaining alertness, adjusting for covariates. Difficulty maintaining alertness “Some Days” and “Most Days/Every Day,” as compared to “Never/Rarely,” were associated a greater risk of all-cause mortality (p<0.01 and p<0.01, respectively). (**B**) Survival curve from the Cox model examining all-cause mortality and napping, adjusting for covariates. Napping “Some Days” and “Most Days/Every Day,” as compared to “Never/Rarely,” were associated a greater risk of all-cause mortality (p<0.05 and p<0.01, respectively). (**C**) Survival curve from Cox model examining all-cause mortality and sleep quality, adjusting for covariates. Sleep quality reported to be “Poor/Very Poor,” as compared to “Good/Very Good,” was associated a greater risk of all-cause mortality (p<0.01). (**D**) Survival curve from Cox model examining all-cause mortality and sleep duration, adjusting for covariates. Sleep duration ≤5 hours, as compared to 7-8 hours, was associated a greater risk of all-cause mortality (p<0.01).

In the fully adjusted Cox proportional hazard models, the risk of all-cause mortality was higher for those participants who reported difficulty maintaining alertness “some days” (HR = 1.49, 95% CI: 1.13 - 1.94, p<0.01) and “most/every day” (HR = 1.65, 95% CI: 1.17 - 2.32, p<0.01, see Figure 2A); for those who reported napping “some days” (HR = 1.38, 95% CI: 1.03 - 1.85, p<0.05) and “most/every day” (HR = 1.73, 95% CI: 1.29 - 2.32, p<0.001, see Figure 2B); those reporting “poor/very poor” sleep quality (HR = 1.75, 95% CI: 1.17 - 2.61, p<0.01, see Figure 2C), and those reporting sleeping 5 or fewer hours per night (HR = 2.38, 95% CI: 1.44 - 3.92, p<0.01 see Figure 2D).

### Relationship between all sleep characteristics and incident dementia and all-cause mortality

Table 5 summarizes results of the Cox proportional hazard models examining all sleep characteristics in a single model and incident dementia (Model A) and all-cause mortality (Model B), after adjusting for confounders. Among the sleep characteristics, only sleep duration ≤5 hours was associated with greater risk of incident dementia (HR = 2.62, 95% CI: 1.48 - 4.64). Among the sleep characteristics, only difficulty maintaining alertness “some days” (HR = 1.42, 95% CI: 1.05 - 1.92), difficulty maintaining alertness “most/every day” (HR = 1.57, 95% CI: 1.05 - 2.33), and sleeping ≤5 hours (HR = 2.07, 95% CI: 1.15 - 3.74) were associated with risk of all-cause mortality.

**Table 5:** Cox model examining all sleep disturbance characteristic and incident dementia (Model A) and all-cause mortality (Model B) (N = 2,812), adjusting for covariates.

	**Model A** **Incident Dementia**^a^				**Model B** **All-Cause Mortality**^b^			
	**HR**	***P*-Value**	**Lower**	**Upper**	**HR**	***P*-value**	**Lower**	**Upper**
Difficulty with Alertness								
Never/Rarely								
Some Days	1.14	0.427	0.83	1.57	**1.42**	**0.022**	**1.05**	**1.92**
Most Days/Every Day	1.08	0.730	0.70	1.67	**1.57**	**0.026**	**1.05**	**2.33**
Nap Frequency								
Never/Rarely								
Some Days	1.04	0.818	0.75	1.45	1.14	0.416	0.83	1.57
Most Days/Every Day	0.99	0.954	0.70	1.41	1.34	0.080	0.97	1.87
Sleep Latency								
<15 minutes								
15-30 minutes	1.21	0.230	0.88	1.66	1.21	0.198	0.90	1.63
>30 minutes	1.44	0.056	0.99	2.10	0.98	0.901	0.67	1.43
Sleep Quality								
Good/Very Good								
Fair	0.76	0.148	0.53	1.10	1.05	0.766	0.76	1.46
Poor/Very Poor	0.82	0.477	0.47	1.42	1.28	0.348	0.77	2.13
Sleep Duration								
7-8hours								
≤5 hours	**2.62**	**0.001**	**1.48**	**4.64**	**2.07**	**0.016**	**1.15**	**3.74**
6-7 hours	0.92	0.624	0.65	1.30	1.06	0.737	0.77	1.45
≥9 hours	1.06	0.779	0.71	1.57	1.23	0.330	0.81	1.87
Snoring^c^								
Never/Rarely								
Some /Most /Every Night	1.67	0.050	1.00	2.80	1.27	0.368	0.75	2.15

Notes: bold indicates significance at the p<0.05 level. ^a^Adjusted models control for demographic characteristics, including age, marital status, race, education, health conditions, and body weight. ^b^Adjusted models control for demographic characteristics, including age, marital status, race, education, health conditions, body weight, and incident dementia. ^c^Due to a small sample size in “Most/Every Night,” the comparison conditions “Some,” Most Nights,” and “Every Night” were combined as the exposure for snoring.

## DISCUSSION

Overall, our findings show a strong relationship between several sleep disturbance and deficiency variables and incident dementia over time. Compared to sleeping 7-8 hours per night, sleeping fewer than 5 hours per night was associated with two-fold greater risk for incident dementia, and routinely taking 30 minutes or longer to fall asleep was associated with a 45% greater risk for incident dementia. We also found associations between sleep disturbance and deficiency variables and all-cause mortality. Specifically, an increased risk of all-cause mortality was observed for those routinely experiencing difficulty maintaining alertness, those reporting routinely napping, those with poor sleep quality, and those reporting sleeping 5 or fewer hours per night.

Whereas previous research on sleep among older adults and risk for incident dementia or all-cause mortality focus on specific sleep constructs, our study prospectively examined a broad array of sleep characteristics and incident dementia and all-cause mortality. We did not observe consistent associations between all sleep characteristics and the two outcomes (i.e., incident dementia and all-cause mortality). For instance, longer sleep latency was associated with incident dementia but not all-cause mortality, yet short sleep duration was associated with both incident dementia and all-cause mortality. Further, in the combined analyses where all sleep characteristics were entered into the same model predicting either incident dementia or all-cause mortality, we found only sleep duration ≤5 hours to be a significant predictor of incident dementia, whereas difficulty maintaining alertness (i.e., “some days,” “most days” or “every day”) and sleep duration ≤5 hours were the only significant predictors of all-cause mortality. Taken together, these results from individual Cox models and the combined Cox models suggest that short sleep duration, after controlling for relevant covariates, is the most important predictor of incident dementia and all-cause mortality.

Given the literature showing a strong association between sleep apnea (a disorder for which loud snoring is the most common symptom) and cognitive impairment [7, 15], it was surprising not to see an association between frequent snoring and either incident dementia or all-cause mortality. Several possible explanations may be hypothesized for this lack of a significant relationship between snoring and either incident dementia or all-cause mortality. First, the wording of the snoring question (“In the last month, how often did you have trouble staying asleep because you snored loudly, or you woke up gasping or choking”) is problematic as it does not account for hearing impairment, which is common among the sample of older adults. Additionally, the question combines several symptoms, which are not necessarily part of the same continuum (i.e., difficulty staying asleep and loud snoring). Furthermore, common questionnaire wording asks for participants to consider others and their reports of the participant and their snoring. By way of example, one common snoring questionnaire asks participants “do you snore loudly, loud enough to be heard through closed doors or your bed-partner elbows you for snoring at night?” [22] Finally, there were few individuals who reported snoring “most nights” or “every night” in the sample. Specifically, 9% of the sample in 2013 reported snoring “most nights” or “every night” and 2% of the sample in 2014 reported snoring “most nights” or “every night.”

Nevertheless, the results from our primary objective in this study highlight an adverse association between sleeping 5 hours or fewer and both incident dementia and all-cause mortality. Our findings are consistent with other cohorts measuring sleep deficiency at baseline and cognitive impairment at follow up [23] and a meta-analysis that also documented a strong association between short sleep and all-cause mortality [20]. The association observed in our study between short sleep (5 hours or less) and incident dementia screening may be understood via the research drawing upon animal models to demonstrate brain toxin removal during sleep [24]. Specifically, research has shown that clearance of Alzheimer’s disease biomarkers, including amyloid-beta plaques or tau proteins [24], takes place at an accelerated rate in the brain during sleep compared to wakefulness, and the rate of buildup of Alzheimer's disease biomarkers is greater during wakefulness than during sleep. These data are consistent with the hypotheses that extended wakefulness and/or sleep deficiency are associated with greater buildup of toxic metabolites and/or impaired clearance of those metabolites, thereby increasing the risk of Alzheimer's disease [25, 26].

It is interesting, however, that our study documented an association between long sleep (9 hours or more) and both incident dementia and all-cause mortality in unadjusted models, but the relationship between long sleep and both incident dementia and all-cause mortality disappeared after controlling for confounders (e.g., age and chronic conditions). In contradistinction, the relationships between short sleep and both incident dementia and all-cause mortality remained significant after full adjustment. Our findings therefore stand in contrast to several meta-analyses that have found associations between both short and long sleep and all-cause mortality in adults [20, 21]. The most parsimonious explanation for the disappearance of the effect of long sleep on dementia and mortality in adjusted models is that the deleterious impact of long sleep is a reflection of underlying disease.

### Limitations and future research

Several limitations and strengths are important to note in the current study. First, our analysis was conducted using questionnaire data that was captured from only approximately one quarter of the NHATS cohort, which was chosen at random in two study years. Without the full sample, we could not apply population weights to produce nationally representative estimates which may have resulted in different findings. Second, sleep data were only available in two study years. While NHATS is a longitudinal dataset and offers rich insights into the nature of dementia development and progression and ultimately all-cause mortality, our analyses were focused on the two years in which sleep questionnaires were administered. Third, several response categories had limited sample size. Specifically, fewer than 10% of the sample reported snoring either “some nights,” “most nights,” or “every night.” This limited our ability to conduct stratified analyses, such as examining the relationship between sleep characteristics and outcomes (i.e., dementia and mortality) by age or by chronic condition, which may have yielded illuminating findings. Fourth, our study examined all-cause mortality as reported by proxy. It would have been a strength if these data could have been obtained by the study using objective reports, such as death certificates. Our study offers numerous ideas for future research. First, there is an urgent need to identify the specific recommendations for improving sleep among older adults. These individuals face an already significant increase in sleep disturbance, yet may benefit from sleep health recommendations more nuanced than those for other categories of adults. Second, although not causal, our data suggest that a new lens for sleep may be important for older adults. Specifically, whereas younger adults are advised to sleep for 7 to 8 hours, our findings show that older adults who report sleeping 6 or more hours may receive a benefit from the standpoint of dementia and all-cause mortality beyond those who sleep 6 hours or less. Third, future research must be undertaken to consider the causal relationship between sleep and incident dementia and all-cause mortality among older adults.

## CONCLUSIONS

Our study offers a contribution to the literature on sleep among aging populations in its assessment of incident dementia and all-cause mortality and a range of sleep characteristics among older adults. According to our findings from Cox proportional hazard models examining each sleep characteristic and outcome, we found that longer time to fall asleep and short sleep duration predicted incident dementia, while short sleep duration, difficulty maintaining alertness, napping, and poor sleep quality predict all-cause mortality. Short sleep duration was a strong predictor of both incident dementia and all-cause mortality, suggesting this may be a sleep characteristic that is important—over and above the other predictors—of adverse outcomes among older adults. Also, future research may consider the development of novel behavioral interventions to improve sleep among older adults.

## MATERIALS AND METHODS

Data from the National Health and Aging Trends Study (NHATS), an annual in-home, computer-assisted, longitudinal, nationally representative survey of community-dwelling Medicare beneficiaries 65 years and older drawn from the Medicare enrollment database, were analyzed. The NHATS data collection began in 2011 with a core interview administered annually. Adults ages 65 and older were sampled from the Medicare enrollment file. NHATS also used proxy respondents for those individuals who were unwilling or unable to complete an interview, a practice which has been shown to reduce attrition bias in longitudinal studies with older adults [27]. Additional information regarding the study’s sampling strategy, design and content are available to the public [28]. All respondents provided consent, and the study protocol was approved by the Johns Hopkins University Institutional Review Board (IRB). Our analysis of the publicly available, de-identified data from NHATS was considered exempt from IRB review.

### Participants

Sleep questionnaires were administered in 2013 and 2014 to a randomly selected subset of the larger NHATS population. In 2013, 27% of the sample was randomly selected to receive the sleep questionnaire (n = 1,575) out of a total 5,799 respondents, and in 2014, 26% of the sample was randomly selected to receive the sleep questionnaire (n = 1,237) out of a total of 4,737 respondents. Participants with dementia at baseline (year 2013) were excluded (n = 202) for a sample of 2,812 with sleep data in either 2013 or 2014. We utilized this sub-sample of NHATS participants that were randomly selected to respond to the sleep supplement in either 2013 or 2014 to understand the relationship between these sleep characteristics, incident dementia, and all-cause mortality in each year leading up to 2018. A flow diagram can be found in the Supplementary Figure 1 section detailing the participants included in this study.

To determine if the NHATS subset analyzed in this manuscript differed from the full cohort, we performed Pearson chi-square tests to examine potential differences in the demographic variables age, marital status, and race between the sub-sample that was included in this analysis and the full sample in each year. We found no difference in marital status (p>.05) or race (p>.05) between those in the sub-sample analyzed in this manuscript and the full sample, but we did find that age varied between the sub-sample analyzed in this manuscript and the full sample (p<0.05). Specifically, those aged 65-69 represented 17% of the full sample and 21% of the sub-sample, those aged 70-74 represented 20% of the full sample and 22% of the sub-sample, those aged 75-79 represented 19% of the full sample and 21% of the sub-sample, those aged 80-84 represented 20% of the full sample and 20% of the sub-sample, those aged 85-89 represented 14% of the full sample and 11% of the sub-sample, and those aged 90 or above represented 11% of the full sample but 6% of the sub-sample. Due to the small differences in these proportions by age, we retained the entire sub-sample in the present analysis.

### Measures

### Sleep characteristics

We examined several characteristics of sleep disturbance. Sleep duration was reported by respondents in hours rounded to the nearest whole number. We created a variable to stratify sleep duration into the following categories: 1) recommended duration (7-8 hours); 2) short sleep duration (6-7 hours); 3) very short sleep duration (≤5 hours); and 4) long sleep duration (≥ 9 hours). Participants marked their response to sleep latency in minutes. Sleep latency responses were categorized into a three-level variable: 1) <15 minutes; 2) 15-30 minutes; and 3) >30 minutes.

Next, a series of sleep characteristics were measured on Likert scales. First, difficulty maintaining alertness was measured with the question “In the last month, how often did you have trouble staying awake at times during the day when you wanted to be awake” on a 5-point Likert scale from “never” to “every day.” Next, sleep quality was measured with the question “In the last month please rate the quality of your sleep” on a 5-point Likert scale from “very poor” to “very good.” Napping frequency was measured with the question “In the last month, how often did you take naps during the day” on a 5-point Likert scale from “never” to “every day.” Finally, snoring was measured with the question “In the last month, how often did you have trouble staying asleep because you snored loudly, or you woke up gasping or choking” on a 5-point Liker scale from “never” to “every night.” We reverse coded all responses so that higher values indicated greater frequency of the sleep parameter (i.e., sleep quality, snoring, napping) and lower values indicated lower frequency of the parameter. All responses to the questions with 5-point Likert scales were transformed into 3-level variables (e.g., difficulty maintaining alertness responses were recoded: 0 was used to indicate “never” and “rarely” responses, 1 was used to indicate “some days,” and 2 was used to indicate “most days or “every day”).

### Screening for incident dementia

To assess cognitive capacity, participants first rated their memory and then performed a memory-related activity (immediate and delayed 10-word recall) [29]. Also, as part of the memory assessment, participants were asked to respond to items related to orientation and perform a clock drawing test to assess executive function [30]. For proxy interviews, the Ascertain Dementia 8-item (AD8), an informant screener for dementia, was administered [31, 32]. A score of 2 or higher on the AD8 is indicative of dementia. Scores on the AD8 and performance on orientation, memory and clock drawing tests were used in our study to form a screening result of either negative (no dementia) or positive (risk for probable dementia). Over the follow-up interval, 321 individuals met the criteria for incident dementia.

### All-cause mortality

The participant’s death was reported to the study personnel by informants during attempts to contact the participant for their annual interview.

### Covariates

In adjusted analyses, we controlled for time-varying covariates including marital status, chronic conditions, body weight, and depressive symptoms. Body weight was reported by participants annually in pounds. Depressive symptoms were measured using the Patient Health Questionnaire-2 which was administered as part of the NHATS [33]. Chronic conditions reported by the sample included self-reported diagnosis of the following conditions: heart attack, heart disease, hypertension, arthritis, diabetes, stroke, and cancer. We included a single variable indicating the number of chronic conditions reported by each individual in the adjusted models. Covariates which did not change over time, including sex and educational attainment, were entered from the baseline interview. Baseline age was also included as a covariate. In models examining all-cause mortality, dementia was also added as a confounder.

### Statistical analyses

We computed descriptive statistics for demographic factors and for sleep characteristics (Table 1). Demographic and health condition data were obtained from the annual NHATS questionnaires in 2013 and 2014. We performed Cox proportional hazards modeling to examine the prospective relationship between sleep characteristics reported at baseline (either years 2013 or 2014) and risk of incident dementia (primary aim) and subsequently all-cause mortality (secondary aim) in the 5 or 4 years of follow-up. Using Cox proportional hazards models, we modeled each outcome (primary: incident dementia; secondary: all-cause mortality), entering the sleep variables individually (Tables 3, 4). Analyses were conducted both without confounders (unadjusted) and with confounders (adjusted). Next, using Cox proportional hazards models, we modeled each outcome, entering all sleep variables simultaneously in the same model while adjusting for covariates (Table 5).

The distribution of the data was assessed to ensure assumptions for all hypothesis testing were met (i.e., proportional hazards). All tests were two-sided with alpha set at 0.05. All analyses were performed in Stata (Version 16, College Station, TX).

## Supplementary Material

Supplementary Figure 1
