# Supplementary figures and images for: Examining sleep deficiency and disturbance and their risk for incident dementia and all-cause mortality in older adults across 5 years in the United States
Source: Aging (Albany NY). 2021 Feb 11;13(3):3254–68. doi: 10.18632/aging.202591 (PMC7906211; doi:10.18632/aging.202591)

## SUPPLEMENTARY FIGURE

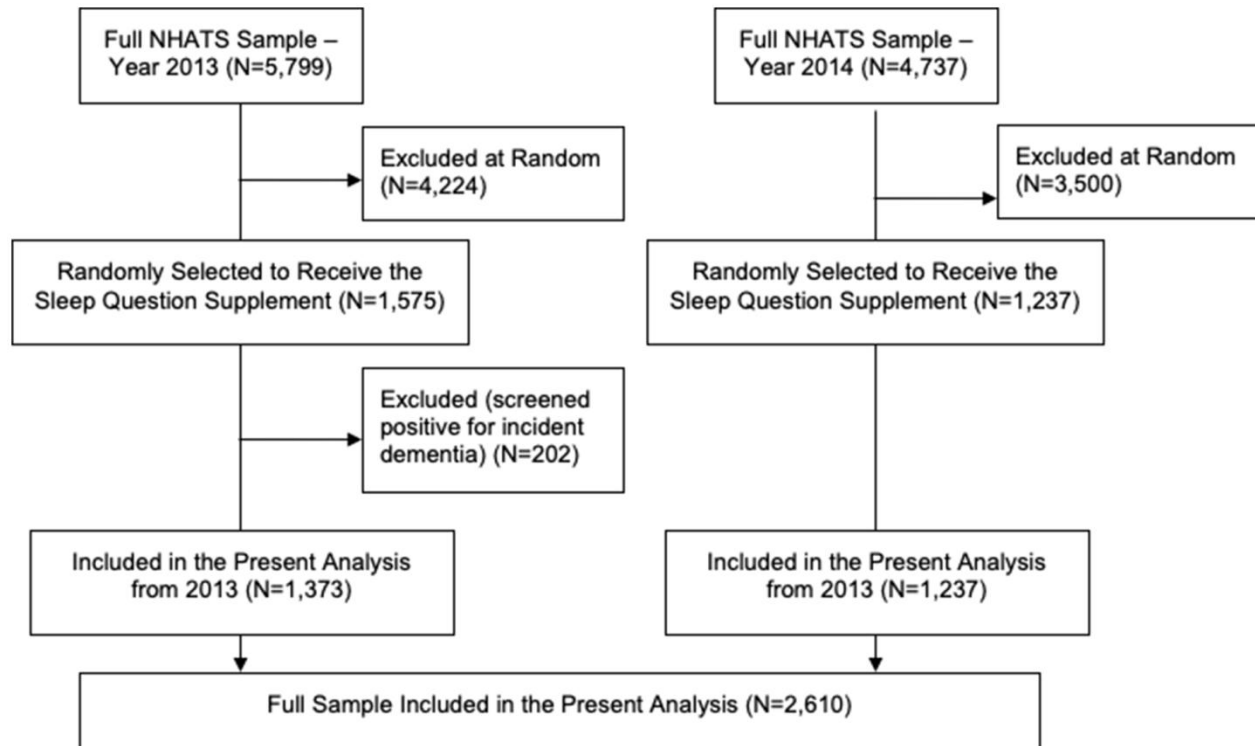

Supplementary Figure 1. Flow diagram outlining study participants.

Supplement: Supplementary Figure 1 [file aging-13-202591-s001.pdf]
